# Advancing the Understanding of Acupoint Sensitization and Plasticity Through Cutaneous C-Nociceptors

**DOI:** 10.3389/fnins.2022.822436

**Published:** 2022-05-10

**Authors:** Xiang Cui, Kun Liu, Xinyan Gao, Bing Zhu

**Affiliations:** Department of Physiology, Institute of Acupuncture and Moxibustion, China Academy of Chinese Medical Sciences, Beijing, China

**Keywords:** acupuncture, hyperalgesia, silent nociceptor, axonal reflex, nociceptor-immune interaction

## Abstract

Acupoint is the key area for needling treatment, but its physiology is not yet understood. Nociceptors, one of the responders in acupoints, are responsible for acupuncture manipulation and delivering acupuncture signals to the spinal or supraspinal level. Recent evidence has shown that various diseases led to sensory hypersensitivity and functional plasticity in sensitized acupoints, namely, acupoint sensitization. Neurogenic inflammation is the predominant pathological characteristic for sensitized acupoints; however, the underlying mechanism in acupoint sensitization remains unclear. Recent studies have reported that silent C-nociceptors (SNs), a subtype of C nociceptors, can be “awakened” by inflammatory substances released by sensory terminals and immune cells under tissue injury or visceral dysfunction. SNs can transform from mechano-insensitive nociceptors in a healthy state to mechanosensitive nociceptors. Activated SNs play a vital role in sensory and pain modulation and can amplify sensory inputs from the injured tissue and then mediate sensory hyperalgesia. Whether activated SNs is involved in the mechanism of acupoint sensitization and contributes to the delivery of mechanical signals from needling manipulation remains unclear? In this review, we discuss the known functions of cutaneous C nociceptors and SNs and focus on recent studies highlighting the role of activated SNs in acupoint functional plasticity.

## Introduction

Acupoints are specific but poorly defined sites in the body responsible for acupuncture therapy. In recent years, the “broad acupoint phenomenon” related to acupoints, the specific and non-specific effects of acupoints, and the qualitative determination of dry-needling trigger points have made determining the nature of acupoints of utmost importance (Melzack et al., 1977; Dorsher, 2008; Li et al., 2015; Langevin and Wayne, 2018). Although considerable effort has been devoted to acupoints identification, the anatomical structures of acupoints remain unclear. Understanding the relation among classic, *Ashi*, trigger, and other extra points and explaining the placebo effect of acupoints and non-acupoints on pain management and other visceral diseases are considered important (Manheimer et al., 2010; Wechsler et al., 2011). Therefore, exploring the intrinsic properties of acupoints will be useful for our understanding of the underlying reasons. Considerable progress has been made in understanding the nature of acupoints, and the results showed a high overlap between acupoints and referred hyperalgesia areas during disease occurrence. Moreover, these overlapped acupoints mainly manifest sensory hypersensitivity, range enlargement, and functional enhancement, namely, acupoints sensitization (Tan et al., 2019). Previous studies revealed that acupoints are related to anatomical structures such as sensory nerves (Kagitani et al., 2005), blood vessels, lymphatic vessels (Lou and Jiang, 2012), connective tissues (Langevin and Yandow, 2002), and tissue space areas (Wang et al., 2010). However, recent studies showed no intrinsic anatomical differences between routine acupoints and adjacent areas (Lou and Jiang, 2012; Mingfu et al., 2013). These results indicate that more attention is required in exploring the underlying neural substrate of acupoints in response to acupuncture manipulation, instead of anatomical structure. Chang et al. (2019) reported that peripheral sensory nerve tissue, but not connective tissue, mediated the therapeutic effect of acupuncture. Direct stimulation of the peripheral sensory nerve produced a similar analgesic effect as acupoint (Li et al., 1998; Kim et al., 2010; Liu et al., 2021), whereas ablated sensory nerve diminished the analgesic effect of acupoint or the modulatory effect on vagal-adrenal axis induced by needling manipulation (Noguchi and Hayashi, 1996; Kim et al., 2010; Liu et al., 2021), indicating that the sensory nerve in acupoint has a vital role in acupuncture effect.

Nociceptors, also known as peripheral sensory fiber endings, are the underlying neural substrate in acupoints and are responsible for the inputs of sensory and acupuncture stimulation. Sensitization of nociceptors induced by external stimulation exhibits a hyper-excitatory state and activation threshold reduction (Pace et al., 2018), also regarded as neuroplasticity. It mediates the mechanism of sensory stimulation transmitting both at peripheral (transduction of the painful stimulus in an electric nerve impulse) and central (transmission, modulation, and perception of the impulse in the spinal cord and brain) levels, which then contribute to the mechanism of sensory sensitization. Hence, we hypothesized that the neuroplasticity plays a key role in enhancing acupoint signals. Nociceptors are mainly divided into A-delta and C nociceptors, which both can be activated by acupuncture innervation in an intensity-dependent manner Several studies revealed that C nociceptors play a key role in acupuncture effect and acupoint sensitization (Zhu et al., 1990; Wang et al., 1997; Zhang et al., 2019). Blockage of peripheral C fiber/nociceptor in acupoint diminished the acupuncture therapeutic effects of antihyperalgesia (Wang et al., 1997; Yang et al., 2013; Zhang et al., 2019) and regulatory effects on the cardiovascular system (Tjen et al., 2005; Zhou et al., 2005; Yuan et al., 2019, 2020). Of note, recent studies elucidated a silent type of C nociceptor “awakened” by inflammatory substances and subsequent conversion into mechanical responses (Gold and Gebhart, 2010), which activated neighboring sensory endings or neurons and were involved in pathological hyperalgesia. Whether activated silent C nociceptors (SNs) are responsible for the enhancing effect of sensitized acupoints *via* augmenting transmission and perception of mechanical signals of acupuncture remains unclear. In this review, we first summarized the research on the intrinsic properties of acupoints based on cutaneous nociceptors, and we discussed the known distribution and physiological functions of cutaneous C nociceptors and SNs and recent studies highlighting the role of awakened SNs in acupoint functional plasticity at the peripheral and spinal levels.

## Acupoint Sensitization and Its Functional Plasticity

In the 19th century, Henry Head observed some visceral diseases that always led to referred pain on the patients’ body surface and summarized the distribution rule of the somatic referred area with different visceral diseases, namely, Head’s zone (Head, 1893, 1894, 1896). Notably, Beissner et al. (2011) described the high-overlap of the Head’s zone and acupoint location; it has also been shown stimulating the sensitized points in the Head’s zone ameliorated the uncomfortable symptoms or enhanced visceral function, similar to the effect achieved at acupoints (James Mackenzie, 1895). Recently, Zhu et al. observed referred pain on the body’s surface under 20 visceral diseases, and their results showed the high overlap of sensitized areas and acupoint location accompanied by sensory hyperalgesia (Shi et al., 2018; Cui et al., 2019; Wu et al., 2019; Qin et al., 2020; Wang et al., 2020; Zhang et al., 2020). Some researchers statistically analyzed the distribution of superficial sensitization points and acupoint locations caused by visceral diseases. These results show that the overlapped rate of heat-sensitive areas and acupoints are 48.76% and 71%∼92% for trigger points and tenderness points, respectively (Melzack et al., 1977; Birch, 2003; Dorsher, 2008; Ben et al., 2012). Furthermore, the same phenomenon is also observed in animal experiments. Through tail intravenous injection of Evans blue, a dye capable of binding plasma proteins and exudate at the body surface in the presence of alteration to its permeability (Kenins, 1981), the exudation points indicate sites with neurogenic inflammation, including sensory-sensitized and -unsensitized points in the body (Wesselmann and Lai, 1997; Kim et al., 2017), the high overlap between the distribution of exudation points and acupoint locations caused by visceral diseases suggests that acupoints are specific points with neurogenic inflammation in the body related to visceral disease-induced somatic referred pain (Rong et al., 2013; He et al., 2017; Kim et al., 2017; Shi et al., 2018; Cui et al., 2019; Wu et al., 2019; Qin et al., 2020; Wang et al., 2020; Zhang et al., 2020). Additionally, acupoints located at the referred area often exhibit mechanical, thermal, itching, and other sensory abnormal accompanied by local skin morphological changes, such as rash, nodules, and other manifestations. Mounting evidence has shown that mechanical hyperalgesia as the most common condition in sensitized acupoint (Cui et al., 2019; Qin et al., 2020; Huang et al., 2021; Xu et al., 2022), which is fully consistent with the concept of “selection of the pain point as an acupuncture point” in the chapter of *Jing Jin* of *Miraculous Pivot*. Hence, the notion of acupoint sensitization was raised by Yu et al. (2015) acupuncture researchers (Rong et al., 2013). However, although acupoint sensitization provides new information to understand the nature of acupoints, the limitation of acupoint sensitization is that acupoints without sensory or performance change could not be included.

Numerous studies showed that the range of acupoint will be enlarged and that its function will also be enhanced. Stimulating the sensitized acupoint exhibits a superior therapeutic effect on the management of chronic musculoskeletal and non-musculoskeletal pain (Wong Lit Wan et al., 2015; Wang et al., 2016; Zucker et al., 2017), bronchial asthma (Xiong et al., 2014), and allergic rhinitis (Lin et al., 2017) in comparison with non-sensitized acupoint, representing the plasticity of acupoint in range and functions, namely acupoint plasticity. In other words, the activated nociceptors, especially C-nociceptors, in sensitized acupoints lead to enhanced signals afferent in reaction to acupuncture manipulation. For example, Li et al. injected mustard oil into ST-25 of rats to mimic acupoint sensitization. Using jejunal mobility as the evaluation index, they observed that simulated ST-25 sensitization could directly inhibit jejunal mobility and enhance the inhibitory effect of electroacupuncture on ST-25 for jejunal mobility (Li et al., 2021). Additionally, Rong et al. (2013) observed that the scope of ST36 and ST37 acupoints expanded after colon injection of mustard oil in rats, and that needling the two sensitized acupoints produced a larger excitatory effect on wide dynamic range (WDR) neurons in the spinal dorsal horn compared with the unsensitized state. Xu et al. observed that acute intestinal mucosal injury resulted in the sensitization of “Xi Qian,” ST36, and ST37. The threshold of stimulating these sensitized acupoints to evoke C-fiber activation of the sciatic nerve was significantly decreased, and more C-fiber discharges were observed (Xu et al., 2015). These studies indicate that various diseases elicited the enhancement of sensitized acupoint function, but the underlying mechanism remains unclear. Since nociceptors mainly involved in responding to and transmitting various sensory stimulations or signals of acupuncture to the spinal and supraspinal levels, more studies are required to explore the role of cutaneous nociceptors in the neurobiological mechanism of acupoint plasticity and acupuncture effect.

## Distribution of the Nociceptors and Their Function

Nociceptors refer to the peripheral sensory terminals distributed in dermal, muscular, cartilage, and visceral tissues, whose cells are located in the dorsal root ganglion or trigeminal ganglion. Physiologically, nociceptors are responsible for the sensory inputs from potential tissue injury and noxious stimulations and transmitting those and other signals, such as acupoint and acupuncture stimulation, to the spinal dorsal horn and supraspinal levels. Sensitization and functional plasticity are the most important physiological characteristics of nociceptors (Gold and Gebhart, 2010), which mainly exists in two forms (Woolf and Salter, 2000): one appears when autosensitization occurs, mainly evidenced by the reduction of excitatory membrane threshold and the elevation of reaction; the other type is the windup phenomenon in the spinal dorsal horn, which mainly occurs due to the increase of firing frequency and prolongation. These two forms respectively mediate peripheral and central sensory sensitizations.

As aforementioned, during acupoint sensitization, mechanical, thermal, and pressure-sensitive pain and changes in scope and function, which are related to local nociceptor activation (Quiroz-González et al., 2017), can be observed. Cutaneous nociceptors are mainly divided into two subtypes (Basbaum et al., 2009), both widely distributed at acupoints (Li et al., 2004; Zhu et al., 2004; Wick et al., 2007). One is derived from medium, myelinated A-delta nociceptors. They mainly respond to high-intensity mechanical and thermal stimuli associated with first pain or acute pain. The other type is derived from small, non-myelinated C nociceptors, related to the generation of second pain or chronic and recurrent pain. Because various types of noxious stimuli, such as mechanical, chemical, and thermal stimulation can activate C nociceptors, it is also known as the C-polymodal nociceptors (Gold and Gebhart, 2010). According to whether they can secrete neuropeptides, classic C nociceptors can be divided into two types: peptidergic [widely distributed in various tissues and deep skin (Plenderleith and Snow, 1993; Bennett et al., 1996; Perry and Lawson, 1998)] and non-peptidergic [related to the skin, mainly distributed in the epidermis (Taylor et al., 2009)]. A current study considered most C-poly nociceptors to be distributed in the skin (Gold and Gebhart, 2010). Besides, a recent paper also demonstrated that C nociceptors not only mediated exteroception (Indicating it can drive reflexive-defensive reactions to prevent or limit injury after detecting external threats) but also contribute to interoception (Sensing the disruption of body integrity and then driving self-caring response toward to the injured region to reduce suffering) (Ma, 2022). Notably, the behaviors like compressing, rubbing, self-scratch, etc., are not only C fiber mediated self-caring behaviors but also related to the origin of acupoints. Hence, we assume that C-fiber acts as a key driver of self-healing and homeostasis regulation under pathological conditions. Further, we suggest that the meaning of acupoint sensitization is not only to warn the body to pay attention to injured tissue and viscera but also to act as a therapeutic point for treatment.

## Activation of the C Nociceptors Mediates Acupoint Sensitization

Recent studies have shown that the activation of C nociceptors mostly relates to the occurrence of acupoint sensitization (Zhang et al., 2019). As previously mentioned, recent studies of acupoint have shown a high overlap between acupoint distribution and plasma protein exudation points after Evans blue-dye injection (He et al., 2017; Kim et al., 2017). Meanwhile, the activation of cutaneous C nociceptors appears to be more related to exudation points (Bharali and Lisney, 1992). Although previous studies have shown that A fiber and C fiber both contributed to acupuncture analgesia (Kagitani et al., 2010), mounting evidence indicated peripheral C fiber/nociceptor in acupoint mainly mediated acupuncture analgesic effect (Wang et al., 1997; Yang et al., 2013), and that the regulatory effect on the cardiovascular system (Tjen et al., 2005; Zhou et al., 2005; Yuan et al., 2019, 2020) and blockade of C fiber/nociceptor using pharmacological strategies impaired acupuncture antihyperalgesic effect (Wang et al., 1997; Yang et al., 2013). Furthermore, C nociceptors also participated in the neural mechanism of acupoint sensitization. In rats with gastric mucosal injury, He et al. (2017) observed that the intensity of immunofluorescence of calcitonin gene-related peptide (CGRP) and substance P (SP) in the skin of sensitized acupoints was significantly elevated than those of unsensitized acupoints. Zhang et al. observed acupoint ST-35 sensitization in the later phase of rats with knee osteoarthritis. They injected retrograde tracer into ST-35 acupoint to label ST-35 related C and A-delta neurons in the dorsal root ganglia (DRG) and then explored the neural mechanism of C and A-delta neurons in acupoint using *in vitro* electrophysiological techniques. They observed that the excitability and hyperpolarization current (*I*_*h*_-current) density of C-type neurons, but not A-delta, increased in ST35 acupoint related DRG neurons (Zhang et al., 2019). Furthermore, they observed that the increase in *I*_*h*_-current of C-nociceptor neurons was mainly related to hyperpolarization function activated cyclic nucleotide-gated channel subtype-2. This study to some extent verified that C nociceptors are directly involved in acupoint sensitization.

According to different responsive characteristics, C nociceptors can be further divided into mechanical, thermal, cold, itch, and other subtypes (Lynn and Carpenter, 1982; Dubin and Patapoutian, 2010; Gold and Gebhart, 2010; Pace et al., 2018). Among them, a C-nociceptor subtype has “silent” characteristics (Hãbler et al., 1990; Feng and Gebhart, 2011; Prato et al., 2017), and its proportional distribution in human skin amounts to approximately 15–20% (Schmidt et al., 1995). Physiologically, this SN does not respond to mechanical stimulation. However, under pathological conditions caused by tissue injury or visceral disease, this type of nociceptor can be “awakened” and become mechanically responsive (Schaible and Schmidt, 1986; Meyer et al., 1991). Previous evidence used mouse colitis as a model to observe the proportion of mechanically insensitive nociceptors dominating distal colons reduced from 27 to 13%, whereas the number of mechanically responsive C nociceptors increased from 34 to 53% (Feng et al., 2012), suggesting that ≥14% of SNs were “awakened” after modeling and became mechanically sensitive. we proposed that the elevated ratio of mechanosensitive nociceptors contributes to enhancing the transmitting of noxious information to the central nervous system, which may be helpful for the body to sense noxious mechanical perception or response to potential tissue injury. TorebjÖrk et al. (1996) observed that SNs has a broader peripheral receptive field. Not only cutaneous but also viscera and joints have a higher distribution ratio (30∼90% in the heart, bladder vessel, rectum, and knee joint) (Schaible and Schmidt, 1988; Hãbler et al., 1990; Pan and Chen, 2002; Feng and Gebhart, 2011; Prato et al., 2017). Recently, the German Vincenzo team has specifically identified SNs, which can be labeled by nicotinic acetylcholine receptor subunit alpha-3 (CHRNA3) and co-expressed with mechanically gated ion channel PIEZO2 (Prato et al., 2017). Physiologically, CHRNA3 labeled SNs are mostly peptidergic C nociceptors distributed in large numbers in the DRG of C1 to S1 segment. Its central endings are mainly projected to lamina I of the spinal dorsal horn, which is the central part of the central mechanism of acupoint functional plasticity. They further observed that PIEZO2 involved inflammatory mediator neurotrophic factor (NGF)-induced mechanosensitivity in CHRNA3^+^ SNs. These results led us to consider whether SNs can be activated by inflammatory substances in sensitized acupoints and thereby be involved in the functional plasticity of acupoints.

Notably, the origin of acupoints is closely related to instinctive behaviors of compressing, pinching, and rubbing specific parts of the body surface. When certain pathological factors cause abnormal superficial or visceral pain, people instinctively seek to use massage, pinching, pressing, and other stimulation methods to alleviate symptoms. As described in *Huangdi’s Canon of Medicine (Huangdi Neijing) and The Great Compendium of Acupuncture and Moxibustion*, acupuncturists need to explore the sensory sensitivity around the acupoint before performing acupuncture, suggesting that sensory aberrances in the change of acupoint’s range were already observed in ancient time. Therefore, we proposed that the awakened mechanosensitive SNs not only contributed to acupoint sensitization but also to amplifying acupuncture therapeutic effect by enhancing the responsive ability to mechanical needling manipulation.

## Predominant Pathological Changes of Sensitized Acupoints Mediated by C Nociceptors: Neurogenic Inflammation

Early studies revealed that neurogenic inflammation is the main pathological characteristic of acupoint sensitization (He et al., 2017; Kim et al., 2017), which is directly related to the activation of C nociceptors (Bharali and Lisney, 1992; Fang et al., 2021). Neurogenic inflammation is the mechanism by which sensory nerve contributes to inflammation (Choi and Di Nardo, 2018), mainly referring to inflammatory symptoms induced by the release of substances from primary sensory nerve terminals/nociceptors. Various cutaneous or tissue injures result in the activation of A-delta and C nociceptors, which then release endogenous inflammatory substances at their axonal ends, including NGF, bradykinin, histamine, and prostaglandin, especially CGRP and SP. Increasing levels of inflammatory substances lead to the recruitment of immune cells, such as mastocytes and immunocytes, which ultimately cause neurogenic inflammation (Richardson and Vasko, 2002; Choi and Di Nardo, 2018; Sorkin et al., 2018). These inflammatory substances constitute the “inflammatory soup,” which interacts with neighboring nociceptors *via* the neuro-immune reaction and augments the cascade reaction. This leads to the proximal area change to sensitization or sensory aberrance (Willis, 1999). Previous studies revealed that small-diameter C sensory neurons act as a key driver in the generation of neurogenic inflammation, especially the subtype of C nociceptor sensitive to capsaicin (Ferrell and Russell, 1986; Holzer, 1988; Richardson and Vasko, 2002). Of note, these increasing inflammatory substances also “awaken” SNs, which then also release inflammatory substances to participate in neurogenic inflammation (Schmelz et al., 2000a). CGRP and SP, released by activated peripheral sensory terminal or nociceptors act as predominated substances in neurogenic inflammation (Choi and Di Nardo, 2018; Sorkin et al., 2018), not only provoking plasma extravasation and edema *via* increasing vascular permeability (Gold and Gebhart, 2010) but also activating and eliciting degranulation of surrounding mast cells and recruitment of immune cells *via* binding specific receptors on immune cells (Riol-Blanco et al., 2014; Kashem et al., 2015; Gupta and Harvima, 2018), ultimately augmenting the local neuro-immune reaction. Most studies in the field of acupoints have shown that neurogenic inflammation is the underlying neural mechanism of acupoints (Wu et al., 2015; He et al., 2017; Kim et al., 2017; Fan et al., 2018). These studies observed that SP and CGRP markedly increased in visceral disease-induced sensitized acupoint. Furthermore, stimulating an acupoint in a healthy state also leads to neurogenic inflammation similar to the histochemical changes in a sensitized acupoint (Wu et al., 2015; He et al., 2017; Kim et al., 2017; Fan et al., 2018). Hence, we hypothesized that sensitized acupoints have a similar therapeutic effect as acupuncture treatment, which can trigger the process of homeostatic regulation through local neuro-immune interaction under neurogenic inflammation; and acupuncture intervention may further augment and promote this process (Zhu, 2019).

## the Underlying Peripheral and Spinal Mechanisms of C Nociceptors Mediated Acupoint Plasticity

As mentioned earlier, peripheral nociceptors act as the first stage in response to various stimuli on the acupoint. Irrespective of the type of exogenous (peripheral stimulation) and endogenous factors (tissue injury or visceral diseases) that act on nociceptors, they will excite nociceptors to produce action potentials in sensory terminals and transform into a sensitized state (activation), leading to a decrease of the activation threshold and increase of stimulation responsiveness. After sensitization, activated nociceptors exert more signals of sensory and needling stimulation transmitted to the supraspinal level, contributing to the functional plasticity of sensitized acupoint. In this part, we discussed the known functions of C nociceptors in hyperalgesia and acupoint sensitization at the cutaneous, DRG (primary sensitization), and spinal (central sensitization) levels.

### Axonal and Dorsal Root Reflexes

Recent studies have suggested that the peripheral mechanism of the neurogenic inflammatory response in pain is predominantly associated with the axonal and dorsal root reflex (DRR) mediated by C nociceptors (Choi and Di Nardo, 2018; Sorkin et al., 2018). These two reflex mechanisms provide necessary conditions for nociceptors to grow “self-consciousness,” which means that various sensory inputs will be integrated by nociceptors themselves before it reaches the first synapse in the central nervous system (Carlton, 2014). This function of nociceptors is a relevant initial point of acupoint functional plasticity.

Due to this special “self-consciousness” adjustment, the sensory sensitization will acts as an early warning sign, and drive the body to attend to the hyperalgesic area and instinctively seek therapeutic massage or medical treatment. However, some scholars further pointed out that the sensitization of mechanical pain is more related to central sensitization, and that peripheral sensitization mainly mediates the occurrence of thermal sensitivity during pathological conditions (Latremoliere and Woolf, 2009).

**FIGURE 1:**
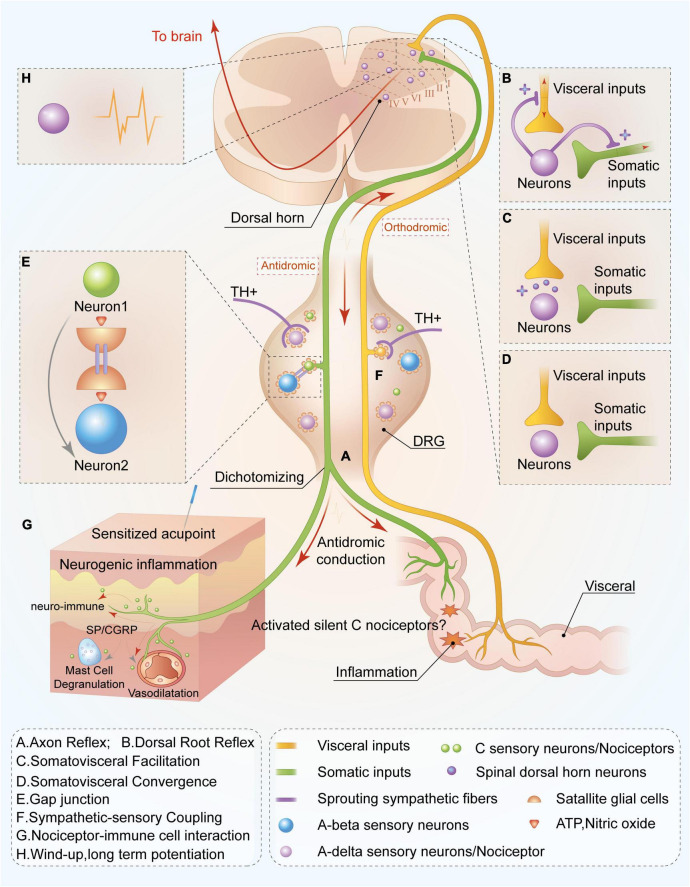
Peripheral and spinal mechanisms of C-nociceptor in acupoint functional plasticity. C nociceptors innervated in the viscera are capable of sensing inflammatory or tissue injury. Noxious signals are orthodromic transmitted not only to spinal and supraspinal levels but also antidromic transmitted to the cutaneous area on the adjacent spinal segments through nociceptors mediating peripheral nerve reflex or the mechanism of somatovisceral convergence and facilitation, resulting in acupoint sensitization at the adjacent spinal segments. Ultimately, increasing levels of C nociceptors distributed in the sensitized acupoints are activated, mediating the functional plasticity of acupoints by enhancing the capacity to respond and deliver acupuncture signaling or peripheral stimulations. Through axon **(A)** and dorsal root reflexes **(B)**, visceral noxious signals are antidromically transmitted to the cutaneous area on the adjacent spinal segments, releasing substance P, and calcitonin gene-related peptide and resulting in neurogenic inflammation and neuro-immune reaction **(G)**. Meanwhile, the activated visceral dorsal root ganglia (DRG) neuron is capable of releasing bioactivators and resulting in sympathetic postganglionic fibers sprouting in DRG **(F)** and simultaneously activating visceral DRG neuron release adenosine triphosphate (ATP) and nitric oxide to sensitize neighboring neurons *via* gap junction **(E)**, leading to more sensory neuron activations. This is involved in the transmission and response of acupuncture treatment. Notably, C silent nociceptors can be “awakened” in the aforementioned inflammatory environment and participate in noxious sensory transmission. In the spinal dorsal horn, noxious inputs from the viscera also lead to excitation of somatic sensory central terminals *via* facilitation **(C)** and convergence **(D)** of somatic and visceral neurons. Moreover, peripheral noxious inputs induce the plasticity of spinal dorsal horn neurons **(H)**, increasing sensory inputs to supraspinal level and improving responsive capacity to peripheral acupuncture application. Overall, this mechanism contributes to the plasticity of acupoint function by improving the capacity to respond to acupuncture treatment.

Axonal reflex is observed when cutaneous or visceral nociceptive stimulation causes excitation of DRG neurons. The generated action potential can not only be transmitted to the spinal cord dorsal horn but also travel through collateral branches in the peripheral bifurcations. The antidromic pathway conducts to its peripheral fiber endings and releases vasoactive substances such as CGRP, SP, and other active substances (including inflammatory factors) at axonal ends, causing neurogenic inflammation (Dubin and Patapoutian, 2010; Choi and Di Nardo, 2018; Sorkin et al., 2018; Figure 1A). Meanwhile, inflammatory substances can activate and awaken SNs, lead them change to mechanosensitive, and enhance thermal responsiveness (Schmidt et al., 1995; Schmelz et al., 2000b), increasing the likelihood of their participation in the pain sensitization process. Animal and human experiments have confirmed that axonal reflexes closely relate to C nociceptors (Jänig and Lisney, 1989; Lewin et al., 1992; Kress et al., 1999). For instance, Lewin et al. (1992) observed that cutaneous C nociceptors participated in the vasodilative exudation caused by axonal reflex. Fang et al. (2021) observed in the rat colitis model that C-nociceptive neurons in the L6-S1 DRG produced bifurcation that simultaneously innervated the viscera and the cutaneous layer, participating in colitis-induced somatic referred hyperalgesia. Additionally, Schmelz et al. (2000a) reported that SNs are directly related to erythema caused by axonal reflex in humans.

In addition to axonal reflex, DRR also contributes to the antidromic conduction of sensory signals from spinal to peripheral levels and participates in hyperalgesia. When nociceptive information activates peripheral nociceptors and subsequently transmits them to the spinal cord horn, it can also antidromically transmit to the peripheral terminals through spinal excitatory interneurons-mediated DRR (Barron Dh, 1938; Toennies, 1938), resulting in inflammatory substances releases, such as SP and CGRP, and the generation of neurogenic inflammation in peripheral tissues (Ferrell and Russell, 1986; Lam and Ferrell, 1989; Rees et al., 1994; Willis, 1999; Figure 1B). Previous studies suggested that sufficient primary afferent depolarization (PAD) acts as a drive for provoking DRR (Rudomin and Schmidt, 1999; Willis, 1999). PAD production and modulation are related to the activation of primary afferent fibers (especially C-fibers) and the involvement of the supraspinal level, including the vestibular nucleus, brainstem reticular formation, red nucleus, midbrain periaqueductal gray, and sensorimotor cortex (Rudomín et al., 1983; Rudomin and Schmidt, 1999; Willis, 1999; Peng et al., 2001; Rudomin et al., 2004). Existing data indicate that although large- and medium- diameter A-beta, and A-delta nociceptors, and small-diameter C nociceptors all relate to the DDR (Willis, 1999), the neurogenic inflammation mediated by DDR and the occurrence of allodynia are mainly related to C nociceptors activation (Weng and Dougherty, 2005).

### Interaction Between Nociceptors and Immune Cells

Mounting evidence has shown that cutaneous immune cells and their mediators could activate peripheral nociceptors, which play a certain role in peripheral neuropathy (Figure 1G). Whether this interaction is involved in acupoint sensitization and functional plasticity is barely understood. The skin comprises two layers: the epidermis and dermis. In the epidermis, the most abundant cell type are the keratinocytes (Nestle et al., 2009); whereas in the dermis, the primary cell type includes myeloid cells, such as Langerhans cells, dermal dendritic cells (dDCs), macrophages, and mast cells, in addition to lymphocyte cells, such as T and B cells (Nguyen and Soulika, 2019). Most of these immune cells release inflammatory molecules to sensitize nociceptors by binding specific receptors. Various stimulations lead to mast cell degranulation which secretes a wide range of mediators including cytokines such as interleukin (IL)-1ß, IL-5, IL-6, and tumor necrosis factor (TNF), tryptase, histamine, serotonin, and NGF, of which all can bind specific receptors on nociceptor endings (Aich et al., 2015; Chatterjea and Martinov, 2015). Tissue injury or infections activate neutrophils releasing inflammatory mediators such as prostaglandin E2, TNF, and interleukins at the site of injury, contributing to nociceptor sensitization (Birklein, 2005; Kanashiro et al., 2020). Increasing cytokines and chemokines released by immune cells act on nociceptors to reduce their threshold and increase their sensitivity to stimuli, contributing to sensory hyperalgesia (Ghasemlou et al., 2015; Ronchetti et al., 2017). Interestingly, these immune cells can be activated by neighboring C nociceptors and participate in physiological and pathological sensory or pain process (Cohen et al., 2019; Lowy et al., 2021). Studies have shown that C nociceptors, activated by various stimuli are capable of releasing SP and CGRP, two important neurotransmitters in the sensitized acupoint, to interact with surrounding immune cells. SP is not only able to elicit dendritic cells to migrate to lymph nodes (Zylka et al., 2005) but can also provoke mast cells degranulation *via* specific binding MrgprB2 receptors on mast cells to mediate the cutaneous inflammatory process (Azimi et al., 2017; Green et al., 2019; Zhang et al., 2021). Furthermore, mast cells have been involved in the antinociceptive effect of noxious acupuncture stimuli on mechanical pain, suggesting that mast cells degranulation contributes to acupoint function. CGRP is capable of eliciting the production of IL-23 from dDCs that drive protective cutaneous immunity (Kashem et al., 2015), exhibiting a vital role in the neuro-immune axis (Assas et al., 2014). Furthermore, cutaneous optogenetic nociceptor stimulation also elevates the levels of dDCs, γδ, αβ T cells and dendritic epidermal T cells in the skin (Cohen et al., 2019; Michoud et al., 2021). Recently, a novel specialized type of “nociceptive Schwann cells” was observed to envelop free nerve endings (Abdo et al., 2019). These nociceptive Schwann cells with extensive processes form a glio-neural end organ with unmyelinated C-nociceptor terminals in the subepidermal layer of the skin, which can respond and transduce noxious mechanical stimuli into electrical signals. The terminals of the C nociceptors sense the noxious signals from Schwann cells and translate these into pain-like behaviors. Overall, this evidence suggests the strong reciprocal interaction between C nociceptors and immune cells in the skin. Whether this association involves acupoint plasticity *via* amplifying signal inputs and augmenting immune regulation of the whole body remains unclear. Moreover, the underlying role needs to be further explored.

### Dorsal Root Ganglia Neuron’s Coupling Activation

The DRG is an important structure in the nociceptive information and proprioception afferent pathway, and various receptions exist in the neuron’s cell body. Current studies have hypothesized that DRG neurons coupling in the activation of satellite glial cells (SGCs) mediates gap junctions and related sympathetic-sensory coupling (Chung et al., 1993; Mclachlan et al., 1993; Huang et al., 2013; Kim et al., 2016; Yamakita et al., 2018). Myriad SGCs distributed around its neurons and noxious stimuli potentially allow these neurons to release various active substances such as nitric oxide and adenosine triphosphate (ATP), to activate surrounding SGCs (Huang et al., 2013; Belzer and Hanani, 2019), which affects neighboring neurons *via* gap junction and leads to the coupling activation of sensory neurons, including hyperexcitation (Schaeffer et al., 2010; Kim et al., 2016; Figure 1E). Furthermore, activated DRG neurons release inflammatory substances, such as NGF, resulting in the sprouting of post-ganglionic fibers that innervate blood vessels supporting to DRG neurons or are distributed in peripheral areas (Mclachlan et al., 1993; Dong et al., 2002). These sprouted sympathetic endings encompass DRG neurons and excrete norepinephrine and ATP to sensitize local neurons (Pertovaara, 2006; Figure 1F). Increased activity increases the transmission of nociceptive information toward the spinal cord dorsal horn which causes central sensitization (Xing et al., 2003).

### Convergence-Facilitation Theory

Somato-visceral convergence and facilitation theory is the foundational mechanisms of referred pain, and it also provides the theoretical basis for understanding acupoint sensitization caused by visceral diseases. Somato-visceral convergence mainly refers to the convergence of somatic and visceral noxious input at the spinal cord and supraspinal level (Ruch, 1946; Roy et al., 1992; Figures 1C,D). Meanwhile convergence-facilitation theory refers to that the central terminals of sensory from viscera and soma synapsed with the same neurons in the dorsal horn, which receives somatic and visceral sensory inputs simultaneously. Noxious input from the viscera leads to the formation of “irritated focal points” within the spinal dorsal horn, which will facilitate non-noxious sensory inputs from the soma and mislead the initial injury area, and finally result in referred pain (Figure 1A). Generally, convergence-facilitation theory explains the importance of its role in the central sensitization of the spinal dorsal horn during pathological conditions. Luz et al. verified the existence of the convergence of soma and viscera through electrophysiological techniques. They observed the convergence of somatic and visceral sensory neurons in the spinal dorsal horn, which also synapsed with the same projection neuron in lamina I of the spinal dorsal horn (Luz et al., 2015), suggesting the anatomical structure in the theory of somatic-visceral convergence and facilitation. However, somato-visceral convergence and facilitation theory suggest a more complex neural integration or interaction in the spinal dorsal horn, which requires more studies to elucidate its underlying role in acupoint sensitization.

### Central Sensitization of the Spinal Dorsal Horn

The spinal cord dorsal horn acts as an integral part of integrating peripheral sensory information and executes central adjustment instructions on the spinal cord. Spinal cord central sensitization functions as an important neural mechanism for secondary hyperalgesia, allodynia, and chronic pain (Pace et al., 2018). The primary central part of A-delta and C-fiber endings synapses with the second-order neurons (such as projection neurons) in lamina I of the spinal cord, which is vital for pain transmission. The portion of project neurons capable of uploading information to the thalamus, periaqueductal gray matter, and parabrachial nucleus, and its other portion transmit information to the rostral ventromedial medulla and participate in the generation and modulation of pain through the descending projection pathway (Todd, 2010). Generally, the central sensitization mechanism at the spinal cord level is related to the following three points (Pace et al., 2018): reduced activation threshold of dorsal horn neurons and enhanced spontaneous activity, “windup” of wide dynamic neurons (WDRs) mediated by activated C nociceptors, and the axons sprouting. Alterations of the synaptic plasticity of dorsal horn neurons, otherwise known as long-time potentiation (LTP), is essential to the aforementioned mechanism. When peripheral A-delta and C nociceptors are activated, an action potential can be orthodromic transmitted toward the central axonal terminal and subsequently release excitatory amino acids (glutamate and aspartate) and neuropeptides (CGRP, SP) at the central endings to activate second-order neurons. Since 80% of projection neurons express NK1 receptor (specific for SP) (Khasabov et al., 2002), NK1^+^ projection neurons activated by SP lead to a windup (acceleration effect) (Hachisuka et al., 2018). Simultaneously, glutamate interacts with *N*-methyl D-aspartate receptors (Khasabov et al., 2002), leading to windup and synaptic plasticity alternation (such as LTP) of spinal dorsal horn projection neurons (Todd, 2010; Figure 1H). This causes central sensitization of spinal and supraspinal levels and finally leads to a reduction in the receptors’ thresholds, and hyperalgesia or allodynia in the body.

Given the spinal mechanism of acupoint plasticity, studies have reported related results about LTP of synaptic plasticity change and hyperactivation of WDR neurons in the spinal dorsal horn. Lv et al. observed the association between colitis-induced somatic referral hyperalgesia (acupoint sensitization) and LTP of dorsal horn neurons using electrophysiological techniques in rat. They showed that colitis modeling resulted in referred mechanical and thermal hyperalgesia in the hind paws, reduced the excitation thresholds of C-fibers, and elevated local field potential in dorsal horns, in comparison with those in the control group, indicating LTP of dorsal horn neurons evoked by C nociceptors participated in colitis-induced referral hypersensitivity (Lv et al., 2019), Rong and Yu investigated the role of WDRs activities in the functional plasticity of sensitized acupoints and the effects of acupuncture. They intracolonic injected mustard oil into rats (Rong et al., 2013) or stimulated the colon by colorectal distension (Yu et al., 2014) and evaluated WDRs activities *via* electrophysiological experiments. Their results showed that the firing rate of WDRs significantly increased after mustard oil intracolonic injections and that the receptive field of WDRs in response to acupuncture stimulation with noxious intensity, not innocuous, in soma also markedly enlarged, indicating the enhanced role of acupoint in WDR activity related to acupoint state and stimulation intensity.

## Summary and Outlook

Nociceptors distributed in the skin over the acupoint are the first to sense the change of microenvironment, acting as a responder in acupoint reacting to acupuncture manipulation, then mediating acupuncture therapeutic effect by delivering the signals to spinal and supraspinal level. Considering the absence of difference in anatomical structure between acupoint and adjacent area, more studies are required to explore the underlying neural substrate in sensitized acupoint, which may provide a better understanding of the phenomenon of broad acupoint, and the similar therapeutic effect of acupoint and adjacent non-acupoint treatment. C nociceptors are widely innervated in the skin and their sensitization contributes to sensory hyperalgesia in pain conditions. Herein, we reviewed the current advances in the study of acupoints and provided a comprehensive perspective on the underlying neural mechanism of C nociceptors mediating acupoint plasticity from the skin, DRG, and spinal levels. Additionally, due to the silent characteristic of SNs and changes in mechanosensitivity after inflammatory conditions, we also highlighted that SNs are possibly strongly associated with the neural mechanism of acupoint sensitization and functional plasticity. Considering that SNs can be labeled by CHRNA3, future studies need to explore the specific contribution in acupoint by manipulating CHRNA3^+^ neurons or nociceptors with chemical and optogenetic approaches. Determining the intricate properties of an acupoint is vital for us to understand the origin of acupuncture, and also provides a new point to optimize the needling targets.

## Author Contributions

XC designed and drafted the manuscript. XC and KL designed and drew the schematic. XG and BZ finished the final version of the manuscript. All authors have read and approved the final manuscript.

## Conflict of Interest

The authors declare that the research was conducted in the absence of any commercial or financial relationships that could be construed as a potential conflict of interest.

## Publisher’s Note

All claims expressed in this article are solely those of the authors and do not necessarily represent those of their affiliated organizations, or those of the publisher, the editors and the reviewers. Any product that may be evaluated in this article, or claim that may be made by its manufacturer, is not guaranteed or endorsed by the publisher.
